# Identification and analysis of breakpoints in “hospital-community-family” cardiac rehabilitation for chronic heart failure patients based on patient journey map: a qualitative study

**DOI:** 10.3389/fcvm.2026.1849187

**Published:** 2026-06-24

**Authors:** Juanqin Shen, Yeping Zheng, Qin Lu, Ting Wang, Xueping Zhang

**Affiliations:** 1 Department of Cardiology, The Second Affiliated Hospital of Jiaxing University, Jiaxing, Zhejiang, China; 2 Department of Nursing, The Second Affiliated Hospital of Jiaxing University, Jiaxing, Zhejiang, China

**Keywords:** cardiac rehabilitation, chronic heart failure, continuity of care, fracture points, patient journey map, qualitative study

## Abstract

**Objective:**

Applying patient journey mapping technology to identify and analyze structural gaps in the “hospital-community-home” cardiac rehabilitation continuum for patients with chronic heart failure, thereby providing empirical evidence for optimizing service linkage.

**Methods:**

Using a purposive sampling strategy, 15 patients with chronic heart failure (NYHA functional class II–III) were recruited from the cardiology department of a tertiary hospital between March and June 2025. Data were collected through semi-structured interviews, and inductive content analysis was employed to code and categorize the data. A patient journey map was constructed to visualize the pain points and gaps in the rehabilitation process.

**Results:**

The study identified nine critical fracture points along the rehabilitation pathway, categorized into three core dimensions: (1) structural fractures (hospital-to-community transition), manifested by the lack of standardized referral processes, discontinuous rehabilitation information transfer, and disparities in access to professional resources; (2) functional fractures (community-to-home transition), manifested by unsystematic home rehabilitation guidance, lack of monitoring methods, and insufficient construction of family support systems; (3) systemic fractures (throughout the entire process), manifested by the absence of multidisciplinary collaboration, inconsistent evaluation standards, and lack of remote support. The formation of these fracture points stems from multiple factors, including uneven allocation of health system resources, inadequate collaboration mechanisms among medical institutions, and insufficient rehabilitation health literacy of patients.

**Conclusion:**

Rehabilitation services for patients with chronic heart failure exhibit significant vulnerabilities in inter-institutional continuity. In response to the identified points of fragmentation, it is recommended to establish a standardized referral system, strengthen family empowerment support systems, and promote the development of intelligent rehabilitation management platforms. These measures aim to bridge service gaps, enhance rehabilitation continuity, improve quality of life for heart failure patients, and reduce readmission rates.

## Introduction

With the increasing burden of cardiovascular disease, cardiac rehabilitation has been confirmed as a key intervention to improve the prognosis of patients with heart failure (1, 2). However, the benefits of rehabilitation services are highly dependent on their continuity (3). In china, although the “hospital-community-home” integrated rehabilitation model is widely advocated, in actual implementation, the transition of patients between different care settings is often accompanied by service interruptions and information loss, leading to low rehabilitation adherence and disruption of treatment continuity, which is detrimental to the long-term control of the disease and improvement of prognosis (4, 5). Patient journey mapping (PJM) technology, as an emerging visualization method, can systematically depict and analyze patients’ experiences, needs, and key nodes throughout the entire disease management process. In recent years, it has demonstrated unique value in chronic disease management and healthcare service optimization (6–9). Based on the above background, this study intends to apply patient journey mapping technology to deeply analyze the connection gaps and their root causes in the “hospital-community-home” rehabilitation chain for patients with chronic heart failure, aiming to provide empirical evidence and strategic recommendations for constructing a seamlessly connected rehabilitation service system for heart failure patients, thereby improving rehabilitation adherence, enhancing quality of life, reducing readmission rates and medical burden, and ultimately achieving precise management and long-term control of chronic heart failure.

## Methods

### Object of study

This study employed a purposive sampling strategy and adhered to the principle of maximum variation. From March to June 2025, patients with chronic heart failure in the cardiac rehabilitation stage were recruited from the department of cardiology of a tertiary hospital. Inclusion criteria were as follows: (1) patients were clinically diagnosed with chronic heart failure and had NYHA functional class II–III; (2) patients were aged 18 years or older; (3) patients had participated in or were participating in a cardiac rehabilitation program in a hospital, community, or family setting; (4) patients had basic smartphone operation ability (note: although this criterion ensures that patients can participate in mobile-health-based rehabilitation research, it may also systematically exclude older patients, those with lower educational attainment, or those from lower socioeconomic backgrounds. These limitations will be discussed in the limitations section). Exclusion criteria were as follows: (1) patients were complicated with serious diseases or complications (such as malignant tumors, serious infections, etc.); (2) patients had auditory or visual disorders that affected communication; (3) patients had a history of mental illness or severe cognitive impairment; (4) patients were unable to complete the basic activities of cardiac rehabilitation. The sample size was determined based on the data saturation principle, meaning that recruitment was halted when the continuous collection of interview data no longer generated new themes or insights. The study protocol was approved by the hospital's ethics committee, and all participants provided written informed consent.

A total of 15 respondents were finally included, and the basic characteristics are shown in Table 1. There were 8 males and 7 females. The mean age was 68.93 ± 6.41 years (range, 55–78 years). The median disease duration was 6 years. There were 9 cases of NYHA class II and 6 cases of class III. In terms of cardiac rehabilitation experience, all 15 patients had experienced hospital-based rehabilitation, 9 had participated in community rehabilitation programs, and only 6 had received formal home rehabilitation guidance.

**Table 1:** Basic information of patients with chronic heart failure (*n* = 15).

Number	Gender	Age (years old)	Duration of illness (years)	NYHA classification	Education	Marital status	Style of residence	Cardiac rehabilitation experience
P1	Male	65	3	Grade II	Elementary school	Married	Live with your spouse	Hospital
P2	Female	72	8	Grade III	Junior high	Married	Live with spouse and children	Hospitals and communities
P3	Male	59	2	Grade II	High school	Married	Live with your spouse	Hospital, community, home
P4	Female	78	12	Grade III	Elementary school	Widowed	Living with children	Hospital
P5	Male	67	5	Grade II	Junior college	Married	Live with your spouse	Hospital, community, home
P6	Female	71	7	Grade III	Elementary school	Married	Live with your spouse	Hospital, community
P7	Male	63	4	Grade II	Junior high	Divorce	Living alone	Hospital
P8	Female	76	9	Grade III	Elementary school	Widowed	Living with children	Hospital, community
P9	Male	74	11	Grade II	Elementary school	Married	Live with your spouse	Hospital, community, home
P10	Female	58	1	Grade II	High school	Married	Live with spouse and children	Hospital, family
P11	Male	69	6	Grade III	Elementary school	Married	Live with your spouse	Hospital, community
P12	Female	73	8	Grade III	Elementary school	Widowed	Living alone	Hospital
P13	Male	62	3	Grade II	Junior college	Married	Live with your spouse	Hospital, community, home
P14	Female	70	5	Grade II	Junior high	Married	Live with spouse and children	Hospital, community
P15	Male	77	10	Grade II	Elementary school	Married	Live with your spouse	Hospital, home

## Research methods

### Formulation of interview outline

Based on the construction principle of patient journey map, semi-structured interviews and descriptive qualitative research methods were used to collect data. The interview outline was developed through four stages: literature review, expert discussion, draft preparation and pre-interview optimization.

Firstly, the research group conducted a systematic search of databases such as the China National Knowledge Infrastructure (CNKI), Wanfang Data Knowledge Service Platform, PubMed, and Medline over the past 10 years for literature related to cardiac rehabilitation, hospital-community-family integrated management, and patient journey maps. The key links, common obstacles, and patient experience evaluation indicators of rehabilitation management for patients with chronic heart failure were then sorted out. Secondly, eight professionals, including cardiologists, rehabilitation therapists, community general practitioners, and nursing experts, were assembled to hold three special seminars. Based on the findings of the literature review and clinical experience, the topic framework of the interview was established. The interview outline was initially divided into four dimensions: disease cognition and diagnosis experience, hospital rehabilitation experience, community rehabilitation connection and experience, and family rehabilitation maintenance and support system. Under each dimension, open-ended main questions and several exploratory queries were formulated to comprehensively capture the experiences, feelings, and needs of patients in the continuous management of cardiac rehabilitation.

The research group selected two patients with chronic heart failure who met the inclusion criteria for a pre-interview. Based on the results of the pre-interview, the outline was revised and refined to form the final interview outline. The main contents were as follows:

1. Disease cognition and diagnosis experience:
  1.1 Please describe your initial symptoms of cardiac discomfort and the subsequent treatment process.
  1.2 How did you learn that you had heart failure? How did the medical staff explain the disease to you?
  1.3 What were your emotional and psychological reactions after learning the diagnosis?
2. Hospital rehabilitation experience:
  2.1 During your hospitalization, did the medical staff introduce the concept and significance of cardiac rehabilitation to you?
  2.2 What kind of rehabilitation training guidance (such as exercise, diet, and medication) did you receive before discharge?
  2.3 How do you think the rehabilitation guidance provided by the hospital was helpful to you? What were the shortcomings?
3. Community rehabilitation connection and experience:
  3.1 After discharge, did the hospital arrange a referral or follow-up plan for you with community medical institutions?
  3.2 What cardiac rehabilitation services have you received in the community? How accessible are these services?
  3.3 What difficulties or obstacles did you encounter during the transition from hospital-based to community-based rehabilitation?
4. Home rehabilitation maintenance and support system:
  4.1 How do you maintain cardiac rehabilitation training at home? What difficulties and challenges do you face?
  4.2 What role does your family play in your recovery? What kind of support do they provide?
  4.3 What do you think an ideal home rehabilitation support system should be like?
5. Breaking points and suggestions for improvement:
  5.1 In the entire process of cardiac rehabilitation, which links do you think have problems of poor connection or service interruption?
  5.2 How do you hope that the hospital, community and family can better cooperate to make your rehabilitation process more coherent and effective?

This interview outline focuses on openness and exploration, and uses a step-by step approach to guide patients to recall and describe the key nodes and turning points in their cardiac rehabilitation journey, with special attention to the transition experience between the three scenarios of hospital-community-home.

### Data collection methods

The researchers screened eligible patients with chronic heart failure rehabilitation through the outpatient appointment system of the hospital's cardiology department. They then contacted these patients, explained the purpose of the study, obtained informed consent, and had the patients sign the consent form before the interview. Fifteen respondents were numbered from P1 to P15, and each interviewee was interviewed for 40–60 min in a quiet room in the hospital. The whole process was recorded with the patients’ consent. The interview was conducted by two researchers who had received training in qualitative research and patient journey mapping (1 heart practitioner and 1 rehabilitation therapist). A semi-structured interview was employed to guide the conversation around the patient's experience of the hospital-community-home cardiac rehabilitation process, focusing on the transition process and the obstacles encountered between different links. The interview encouraged patients with relevant experience to express their true feelings, and at the same time, non-verbal reactions (e.g., expressions and emotions) were recorded. When 3 consecutive patient interviews failed to yield new themes or insights, data saturation was judged, and data collection was terminated. All interview data were transcribed into text and used as the basic data for the analysis of breaking points.

### Data sorting and analysis

The content analysis method was used to systematically analyze the interview data. The recordings were transcribed verbatim into text data within 24 h after the interview and were verified by the respondents to ensure the authenticity and accuracy of the content. Use NVivo 12.0 qualitative analysis software to code the data, and strictly follow the three-level coding procedure: (1) open coding (first-level coding): two researchers who have received qualitative research training independently read the content, extract initial codes related to the rehabilitation experience, and resolve disagreements through discussion and judgment by a third researcher. (2) Axial coding (second-level coding): summarize the initial codes into more general sub-themes (e.g., “lack of referral information”, “difficulty in home monitoring”). (3) Selective coding (third-level coding): refine the core themes and construct a “hospital-community-family” rehabilitation journey framework.

Breaking points were identified based on the following criteria: (1) patient-reported experiences of service interruption or poor connectivity; (2) lack or distortion of information transmission between different links; (3) a noticeable gap between patients’ needs and available services; (4) nodes where patients’ emotional experiences show significant negative changes. For each breaking point identified, the research team further analyzed its root cause, influencing factors, and possible improvement directions. After the preliminary analysis, the research team invited 3 interviewed patients and 2 medical professionals who were not involved in the preliminary analysis to review the journey map to verify the accuracy of the breaking points and optimize them based on feedback, ensuring the authenticity, completeness, and practicality of the research results. Through this process, the “hospital-community-family” journey map and breaking point analysis report for cardiac rehabilitation in patients with chronic heart failure were finalized.

## Results

### Construction of patient journey map framework

**Figure 1:**
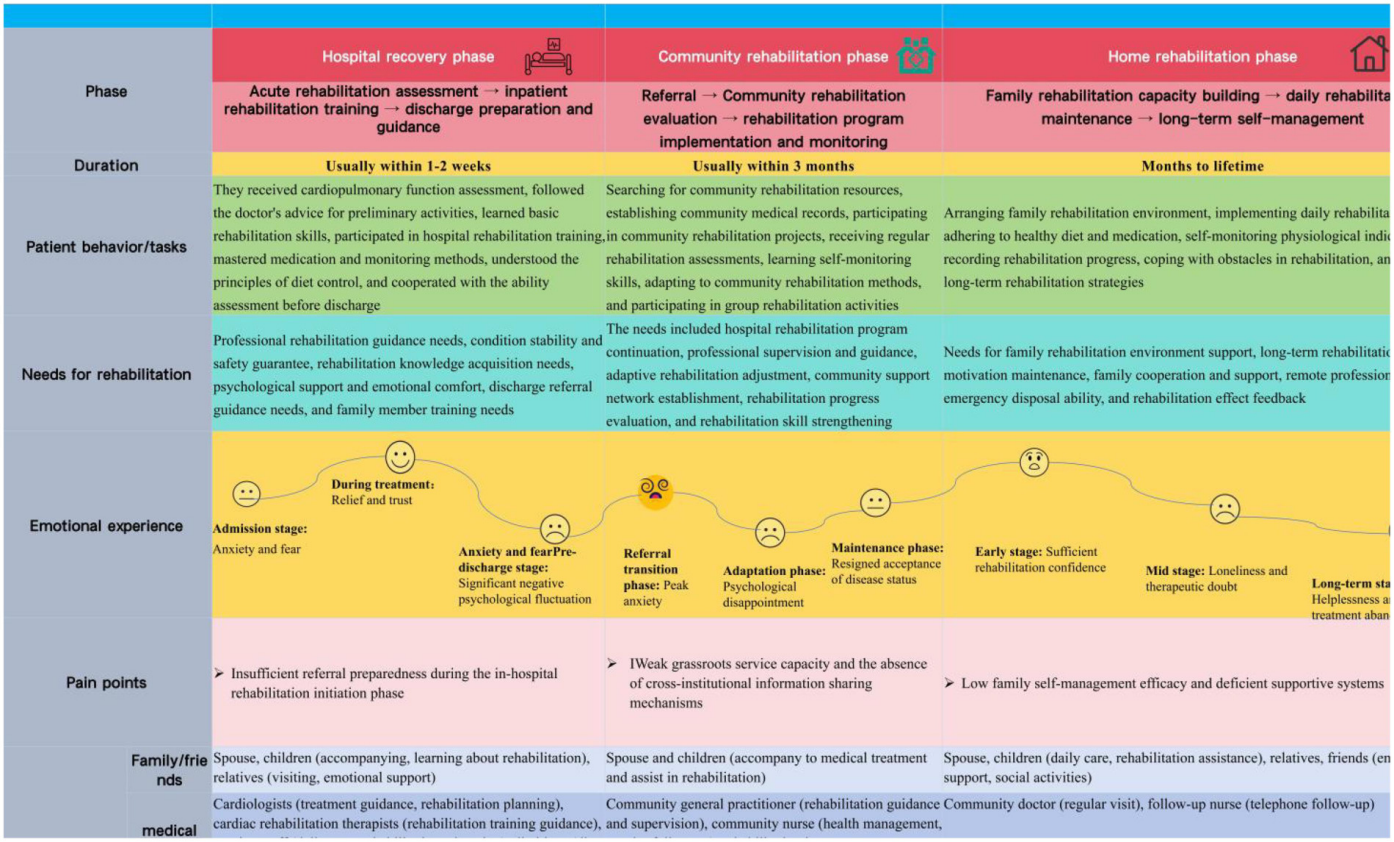
Journey map of patient breakpoints in cardiac rehabilitation for chronic heart failure patients—“hospital-community-family” model.

This study involved a total of 15 interviews. Based on the coding results, the research team adopted a four-dimensional framework—“timeline–touchpoints–pain points–emotional curve”. The horizontal axis was divided into three stages according to the rehabilitation trajectory: “hospital-community-home”. The vertical axis integrated “rehabilitation tasks (touchpoints),” “rehabilitation needs,” “emotional experiences (positive/negative fluctuations),” and “pain points (breaking points)”. Finally, the content of the map was finalized through expert consultation. Analysis of the emotional curve revealed that patients’ emotions experienced significant negative fluctuations (anxiety and confusion) at two key junctures: “discharge referral” and “early home phase” (Figure 1).

### Identification and analysis of breaking points in cardiac rehabilitation

Through an in-depth analysis of the patient journey map, the research team identified nine key breakpoints in the “hospital-community-home” continuum of cardiac rehabilitation for patients with chronic heart failure. These breakpoints were categorized into three core dimensions, and a systematic analysis was conducted on the characteristic features and underlying causes of each breakpoint.

### Structural break (transition period from hospital to community)

The disruption in this dimension is primarily reflected in the absence of systems and procedures, resulting in patients “losing contact” when transferring between institutions.

1. Lack of a standardized referral process. Ten patients reported that they did not receive clear arrangements for community-based rehabilitation referrals upon discharge, resulting in interruptions in their rehabilitation treatment. For example, P2 said: “When I was discharged, the doctor only told me verbally that I could continue rehabilitation in the community, but didn’t specify which community hospital I should go to, nor did they provide a referral form. I had no idea how to proceed on my own.” P8 added: “There seems to be little communication between the hospital and the community. After discharge, I contacted several community hospitals, but none of them could clearly explain how I should continue my rehabilitation given my specific condition.”
2. A disconnect in the rehabilitation information flow. The hospital and community information systems are not interconnected, leaving community doctors unable to access patients’ inpatient treatment and rehabilitation baseline data. As a result, patients are forced to recount their medical histories repeatedly and face the tedious process of undergoing redundant assessments. P11: “The community doctor has absolutely no idea what treatments I received at the hospital. Every time I go there, I have to retell my medical history all over again—it feels like there's zero communication between them and the hospital.” P5: “When I went to the community clinic, I found out they couldn't even see my rehabilitation records from the hospital. Each time, they have to start the assessment from scratch, which is a huge waste of time.”
3. Disparities in access to professional rehabilitation resources. There are significant differences between hospitals and communities in terms of rehabilitation facilities and staffing. P14: “Hospitals have professional rehabilitation therapists and fully equipped facilities, but community settings are much more rudimentary—there’s only one general practitioner responsible for all patients with chronic diseases, and there’s absolutely no specialized cardiac rehabilitation guidance.” P6: “There are too few rehabilitation facilities in the community; some basic monitoring equipment is even missing. I’m afraid to do vigorous exercise there.” P3: “The rehabilitation prescriptions issued by the hospital aren’t adjusted by anyone in the community. My condition has changed, but the community doctor says they don’t dare to alter the hospital's plan arbitrarily.”

### Transition from community rehabilitation to home rehabilitation is broken

The rupture in this dimension manifests as a lack of rehabilitation capacity, leaving patients unable to maintain their recovery once they return home.

1. The home rehabilitation guidance is unsystematic. Eight patients reported that their communities have not provided a systematic home rehabilitation program. P1–P6 and P14: “The community doctor just briefly advised me to keep exercising at home, but didn’t tell me specifically how to do it or clarify the frequency and intensity.” P9: “I’ve been relying entirely on my own trial-and-error approach to home rehabilitation—no one has taught me exactly how to do it safely and effectively.”
2. Lack of home-based rehabilitation monitoring tools. Patients lack effective tools and methods for monitoring their health status at home, making it impossible for them to receive timely professional feedback. P1, P5, and P10: “It’s okay to measure blood pressure at home, but I don’t know what heart rate range I should aim for during exercise, and no one has told me how to determine whether my exercise is appropriate.” P15: “I’m doing rehabilitation at home by myself, with no one supervising me. I’m not sure if I’m doing it correctly, and I’m also worried about what to do if something goes wrong.”
3. Vulnerable family support system: family members lack relevant training and are unable to provide effective supervision. P4: “My daughter is very busy with work and can’t always stay by my side to help me with rehabilitation, but she doesn’t know how to assist me either.” P5: “At first, I could still stick with it, but after a while I started to slack off—no one’s there to keep me on track, and I just can’t seem to motivate myself anymore.” P8: “My family cares about me a lot, but they don’t really understand how cardiac rehabilitation works in detail. Sometimes they even end up stopping me from moving altogether, worried that I’ll get too tired.” P15: “I’m worried that doing rehabilitation at home might pose risks. What if something urgent happens? Who’d be able to help me in time?”

### Systemic fracture (running throughout the entire process)

The rupture in this dimension reflects a deep-seated lack of integration within the service system.

1. Insufficient multidisciplinary team collaboration. As a comprehensive intervention model relying on multidisciplinary collaboration, cardiac rehabilitation suffers from poor communication and coordination among different specialties during actual implementation. P5: “When I was in the hospital, doctors, nurses, nutritionists, and rehabilitation therapists worked together to help me develop a plan. But once I moved to the community, only the general practitioner was left, and many professional recommendations couldn’t be carried forward.” P13: “It feels like each doctor just focuses on their own area of expertise. My medication, diet, and exercise plans aren’t uniformly coordinated—and sometimes they even contradict each other.” P14: “Every time I go to a different place, doctors and nurses give me bits and pieces of rehabilitation knowledge, but no one provides me with a complete rehabilitation guide.”
2. Assessment standards and data silos: assessment standards vary across different stages, and data cannot be shared. P3: “The assessment forms used by the hospital are completely different from those used in the community—there’s no way to compare whether I’ve actually made any progress.” P9: “Each place has different criteria for assessing my condition. The hospital says I’m recovering well, but the community doctor says I still need more improvement—and I myself am confused about exactly where I stand in terms of recovery.”
3. A remote rehabilitation guidance system is lacking. There is a shortage of an effective telemedicine support system, making it impossible to provide patients with continuous, professional rehabilitation guidance. P6: “Every time I go to the community center or return to the hospital for a follow-up visit, I have to re-measure all my indicators from scratch—seems like none of my previous records are shared.” P7: “The community hospital where I live is a bit far away, and it’s especially inconvenient to go out in winter. It would be great if I could receive guidance from a doctor via video call on my phone.” P10: “Sometimes when I encounter problems during rehabilitation and want to consult someone, I don’t know who to turn to. Calling the hospital is often difficult to get through, so I’m left to guess and figure things out on my own.” P12: “Every time I go to a different hospital, I have to retell my medical history and undergo new examinations all over again—as if my medical records aren’t shared at all.”

## Discussion

### Visual analysis of rehabilitation breakdown characteristics based on the patient journey map

This study innovatively applies PJM technology to visually represent the rehabilitation experiences of patients with chronic heart failure along the “hospital-community-home” continuum. Unlike previous studies that relied solely on interview text analysis, the PJM technology constructs a four-dimensional model—comprising “time, touchpoints, emotions, and pain points”—which not only clearly outlines the overall trajectory of patient recovery but also vividly reveals the specific spatiotemporal nodes where service disruptions occur and their dynamic impact on patients’ psychological experiences. The study finds that patients’ emotional curves exhibit a significant “sharp drop-off” during the transition period from hospital discharge to community-based care. This phenomenon underscores the detrimental effects of discontinuity in service continuity on patients. The disruption of patient safety. Specifically, the ruptures identified in this study are not merely a deficiency in a single dimension; rather, they exhibit a complex interplay among structural, functional, and systemic dimensions. Structural ruptures manifest as “hard flaws” in institutions and processes, functional ruptures reflect “soft vulnerabilities” in execution and capability, and systemic ruptures highlight “weaknesses” in top-level design and technological support. This multidimensional nature of ruptures places heart failure patients under significant adaptation pressure during cross-institutional referrals and home-based self-management, thereby undermining their adherence to rehabilitation protocols.

### Analysis of the causes of fractures from the perspective of continuous management

This study finds that although the “hospital-community-family” model has been proposed, significant shortcomings and substantial gaps remain across three key dimensions: clinical continuity (coherence of information and treatment plans), organizational continuity (referral and collaboration), and relational continuity (physician-patient and family support) (10). These multiple discontinuities collectively contribute to a decline in cross-level rehabilitation service quality, insufficient patient adherence to rehabilitation programs, and suboptimal long-term rehabilitation outcomes—issues that are increasingly evident in practice (11, 12).

First, the breakdown of organizational continuity is the core cause of structural fragmentation. An ideal rehabilitation system should be built on the foundation of tiered medical care and two-way referrals (13, 14). However, this study reveals that, due to the absence of standardized referral pathways and mechanisms for aligning interests, higher-level hospitals and primary care institutions often operate as “information silos”. Upon discharge, patients receive only verbal advice without a formal referral document, leaving them effectively “disconnected” during transitions between institutions. This lack of organizational collaboration directly obstructs rehabilitation services at the “last mile”—the critical stage where patients transition from hospital to home.

Second, the fragmentation of clinical continuity exacerbates functional discontinuity. Cardiac rehabilitation is a comprehensive intervention involving exercise, medication, nutrition, psychological support, and smoking cessation, requiring high consistency in assessment criteria and intervention protocols across all stages (2, 15). Yet, in our interviews, patients reported inconsistent assessment tools between community and hospital settings, making it impossible to seamlessly continue their rehabilitation prescriptions. Moreover, the absence of a standardized “rehabilitation handover form” forces patients to become intermediaries in information transfer—a situation that not only increases their cognitive burden but also undermines the seamless nature of care delivery. Such discontinuities in clinical information and the lack of professional guidance leave patients returning home in a state of “no clear guidance and no reliable reference,” rendering home-based rehabilitation largely superficial and thus creating functional fragmentation.

Finally, the fragility of relational continuity leads to the collapse of the support system. Continuous rehabilitation involves not only the physician-patient relationship but also the support provided by family caregivers (16). Our study shows that, currently, family members serving as informal caregivers are not effectively integrated into the rehabilitation support system; they lack both the necessary skills training and emotional support. When patients return home from the highly supervised hospital environment and face complex rehabilitation tasks, their fragile family support systems are prone to collapse, further intensifying uncertainty and risks throughout the rehabilitation process.

### Optimization strategies for cardiac rehabilitation services based on the patient journey map

Based on the visual analysis of the aforementioned breakpoints, this study proposes the following targeted optimization strategies to build a seamlessly connected cardiac rehabilitation service system. (1) Establish a standardized referral and information-sharing mechanism to bridge structural gaps (17). It is recommended to leverage the medical consortium platform to set up a mandatory, structured electronic referral system. Referral forms should not only include diagnostic information but also cover core rehabilitation protocols, exercise tolerance assessment results, and relevant precautions, ensuring that community physicians can “take over effectively and manage well”. At the same time, it is essential to streamline communication and integration between hospitals breaking down information barriers within the community and achieving interoperability of rehabilitation data are key to reshaping organizational continuity. (2) Empowering families and communities to bridge gaps in functional continuity. To address the current gap in family-based rehabilitation guidance, we recommend developing a standardized “cardiac rehabilitation family toolkit” (18, 19). This toolkit could include simple monitoring devices (such as scales and blood pressure monitors), visualized rehabilitation instruction videos, and emergency contact cards. By using standardized tools to replace direct on-site supervision by professionals, we can reduce the difficulty of implementing home-based rehabilitation. In addition, training for family caregivers should be strengthened to enhance their rehabilitation management skills and solidify the family’s role as the primary setting for rehabilitation. (3) Exploring an “internet plus” remote monitoring model to break through systemic bottlenecks (18). Given patients’ urgent need for medical support that transcends geographical boundaries, exploring a remote monitoring model based on mobile healthcare is highly relevant and practical. Although this study did not evaluate specific platforms, the patient journey map clearly highlights the strong desire for real-time monitoring and feedback. In the future, we could explore integrating wearable devices with AI-powered early warning systems to provide patients with round-the-clock rehabilitation support, thereby addressing the systemic challenges of insufficient and unevenly distributed professional resources.

## Conclusion and limitations

This study innovatively applies the patient journey mapping technique to objectively and visually represent the subjective experiences of patients with chronic heart failure. Unlike traditional qualitative interviews, this study employs a combined analysis of emotional curves and touchpoints to precisely identify three critical breakpoints in the transition from “hospital” to “community” and then to “home”. Although limited by sample size and single-center origins, this study clearly reveals that gaps in referral processes, lack of family support, and insufficient use of information technology are the core bottlenecks leading to interruptions in rehabilitation, providing targeted areas for subsequent intervention design.

At the same time, this study has certain limitations: the sample size is relatively small and drawn from a single medical center. Future studies could expand the sample scope to include patients from different regions and medical institutions of varying levels, thereby obtaining more representative data. Secondly, we adopted “having the ability to operate a smartphone” as an inclusion criterion. While this aligns with the trend toward digital rehabilitation, it also means that we may have systematically excluded the most vulnerable patient groups—those with lower socioeconomic status or advanced age—precisely the population that is most likely to suffer from disruptions in rehabilitation services. This selection bias reminds us that future solutions cannot rely solely on technology; rather, they must continue to maintain human support in offline settings. Building on the findings of this study, the research team will subsequently conduct targeted intervention studies to validate the practical effectiveness of strategies aimed at addressing these breakpoints, thus providing a stronger scientific foundation for establishing a coherent and efficient rehabilitation management system for heart failure patients.

## Data Availability

The data analyzed in this study is subject to the following licenses/restrictions: The datasets generated and/or analyzed during the current study are not publicly available due to the sensitive nature of the qualitative data (interview transcripts), which could compromise participant privacy and confidentiality. However, relevant anonymized data excerpts supporting the findings are included within the article. Reasonable requests for further data access can be directed to the corresponding author. Requests to access these datasets should be directed to Juanqin Shen, 15268341093@163.com.
